# The *Salmonella *Pathogenicity Island (SPI) 1 contributes more than SPI2 to the colonization of the chicken by *Salmonella enterica *serovar Typhimurium

**DOI:** 10.1186/1471-2180-9-3

**Published:** 2009-01-06

**Authors:** Yakhya Dieye, Keith Ameiss, Melha Mellata, Roy Curtiss

**Affiliations:** 1 The Biodesign Institute, Center for Infectious Diseases and Vaccinology, Arizona State University, Tempe, Arizona 85287, USA; 2 School of Life Sciences, Arizona State University, Tempe, Arizona 85287, USA

## Abstract

**Background:**

*Salmonella enterica *serovar Typhimurium (Typhimurium) is an important pathogen that infects a broad range of hosts. In humans, Typhimurium causes a gastroenteritis characterized by vomiting, diarrhea, and abdominal pains. Typhimurium infection occurs mainly through the ingestion of contaminated food including poultry, pork, eggs, and milk. Chickens that are asymptomatic carriers of Typhimurium constitute a potential reservoir for infection. The type three secretion systems encoded by *Salmonella *pathogenicity islands (SPI) 1 and 2 are major virulence factors of *Salmonella*. However, only a few studies have investigated their role during the infection of chickens.

**Results:**

We have taken a mixed infection approach to study the contribution of SPI1 and SPI2 to the colonization of the chicken by Typhimurium. We found that SPI1 contributes to colonization of both the cecum and spleen in the chicken. In contrast, SPI2 contributes to colonization of the spleen but not the cecum and, in the absence of SPI1, inhibits cecal colonization. Additionally, we show that the contribution of SPI1 in the spleen is greater than that of SPI2. These results are different from those observed during the infection of the mouse by Typhimurium where SPI2 is the major player during systemic colonization.

**Conclusion:**

The co-infection model we used provides a sensitive assay that confirms the role of SPI1 and clarifies the role of SPI2 in the colonization of the chicken by Typhimurium.

## Background

*Salmonella enterica *is a gram-negative enteric bacterium that comprises about 2500 serovars [1]. While some have a restricted host range (e.g. the serovars Typhi and Pullorum are restricted to humans and chickens, respectively), most of the *S. enterica *serovars can infect a broad range of warm-blooded animals and humans. *S. enterica *infects its hosts by the oral route and primarily causes two types of disease: a gastroenteritis characterized by the development of bacteria in the intestinal tract [2], and typhoid fever that results from the invasion of the systemic compartment [3]. Typhoid fever is a serious health issue in developing countries [4] but is rare in the Western world. In contrast, *Salmonella *gastroenteritis is an important concern worldwide. Food products, including poultry, pork, egg, and milk constitutes an important source of *Salmonella *infection in humans [5]. *Salmonella enterica *serovar Typhimurium (will be referred to hereafter as Typhimurium) is a broad host range serovar that infects humans, cattle, mice, and chickens, and is one of the major causes of food-borne human salmonellosis [6,7].

Typhimurium remains an important concern to the poultry industry [8] causing a systemic infection in newly hatched chicks, often resulting in death [9]. In older birds infection by Typhimurium leads to an asymptomatic carriage state with colonization of the digestive tract and continuous shedding [10,11]. These healthy carrier birds constitute a risk of contamination of newly hatched chickens, as well as the food chain leading to both important economic losses and potential harm to human consumers.

The pathogenesis of *Salmonella *has been extensively studied in the mouse [12]. In susceptible mice, *Salmonella *causes an acute systemic disease with limited intestinal manifestations [13]. Recently, a model of *Salmonella *enterocolitis has been developed in streptomycin-treated mice [14]. Studies using these mice and other animal models of *Salmonella *diseases have yielded substantial data about the molecular players involved at different levels. The *Salmonella *pathogenicity islands (SPIs) 1 and 2 are two major virulence determinants of *S. enterica*. They encode type III secretion systems (T3SS) that form syringe-like organelles on the surface of gram-negative bacteria and enable the injection of effector proteins directly into the cytosol of eukaryotic cells [15,16]. These effectors ultimately manipulate the cellular functions of the infected host and facilitate the progression of the infection. SPI1 and SPI2 play several roles in different organs within the host. SPI1 primarily promotes the invasion of non-phagocytic intestinal epithelial cells and the initiation of the inflammatory responses in the intestines [17,18]. It is also involved in the survival and persistence of *Salmonella *in the systemic compartment of the host [19-21]. The first characterized role of SPI2 was its ability to promote *Salmonella *survival and multiplication in phagocytic cells that constitute the main reservoirs for dissemination of the bacteria into systemic organs [16]. SPI2 also plays an important role in the intestinal phase of *Salmonella *infection in mice [17,22,23].

**Figure 1:**
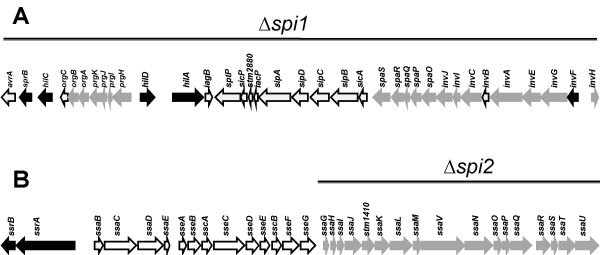
**Genetic organization of SPI1 (A) and SPI2 (B)**. The genes encoding structural proteins are in grey, and the genes that code for transcriptional regulators are in black. The deletions are represented by the black line above the graphs.

The regulation of SPI1 and SPI2 gene expression involves numerous transcriptional regulators located both inside and outside these pathogenicity islands. The regulation of SPI1 is particularly complex. SPI1 encodes for the five regulators HilA, HilC, HilD, InvF, and SprB (Figure 1). The first four of which are involved in regulatory pathways that lead to the activation of SPI1 genes and of genes encoding T3SS effectors located outside SPI1. In contrast to SPI1 the regulation of SPI2 genes is simpler with the SsrAB two-component system being the only transcriptional regulator encoded within SPI2 that activates the expression of SPI2 genes and of genes encoding T3SS effectors located outside SPI2. Interestingly, SPI1 regulators can regulate SPI2 genes. These include HilA that binds and represses the promoter of *ssaH *[24], and HilD that binds and activates the promoter of the *ssrAB *operon [25]. In contrast, SsrAB has never been shown to act on the expression of SPI1 genes.

Few studies have investigated the role of SPI1 and SPI2 during the infection of chickens. In studies using Typhimurium, two approaches have provided data about the roles of SPI1 and SPI2. The first approach compared colonization in chickens by infecting with single strains and enumerating colonies from internal organs. Porter and Curtiss [26] found that mutations in structural genes of the SPI1 T3SS resulted in a reduction of the colonization of the intestines in day-old chickens. Jones *et al*. [27] generated strains with deletions of *spaS *and *ssaU*, genes that encode structural proteins of the SPI1 and SPI2 T3SS respectively, and compared their ability to colonize the cecum and liver in one-day and one-week old chickens to that of wild type. They concluded that both SPI1 and SPI2 play major roles in both the intestinal and the systemic compartments, with SPI2 contributing more than SPI1 in both compartments. The second approach screened random transposon libraries for reduced recovery from the chicken gastrointestinal tract through cloacal swabbing. Turner *et al*. [28] analyzed a library of 2,800 mutants for intestinal colonization in chickens. Among the mutants that showed reduced intestinal colonization they found one in which the SPI1 gene *sipC *was inactivated. No mutations in SPI2 genes were identified in this screen. Morgan *et al*. [29] screened a library of 1,045 mutants in chickens and found two mutations in SPI1 genes and one in a SPI2 gene that led to a reduction in colonization ability. The SPI1 mutants were unable to be recovered from 50% or 100% of the day old birds tested, while the single SPI2 gene was unable to be recovered in only 33%. In this study fourteen strains with mutations in SPI1 and fifteen strains with mutations in SPI2 did not show any defect in colonization. The authors of these two studies concluded that SPI1 and SPI2 play a marginal role in the colonization of chicken intestines by Typhimurium.

To gain better insight in the role of these important virulence factors we have taken a different approach. First, we performed mixed infections in which the strains that are being compared (the wild type and a mutant, or two different mutants) are co-administered. This approach more directly addresses the contribution of SPI1 and SPI2 by decreasing the animal to animal variations inherent in such studies and giving us the ability to test the fitness of two mutants directly against each other. Second, we used one-week-old chicks that are known to be resistant to acute infection by Typhimurium [10,11] allowing us to follow the effect of the studied mutations over a relatively long period of time. Third, we used mutants in which the entire SPI1 and/or the entire structural operon of SPI2 are deleted (Figure 1). This inactivates all the genes involved in both SPI1 and SPI2 T3SS apparatus synthesis and prevents the action of SPI1 regulators on SPI2 gene expression. Using this approach, we compared the colonization of the wild-type to that of each of the mutants.

We report here that SPI1 contributes to the colonization of both the cecum and spleen of the chicken. In contrast, SPI2 contributes to colonization of the spleen but not the cecum and, in the absence of SPI1, inhibits cecal colonization. Additionally, we show that the contribution of SPI1 in the spleen is greater than that of SPI2. These results differ from those observed during the infection of mice by Typhimurium, where SPI2 plays a major role during systemic colonization.

## Results

To assess the roles of SPI1 and SPI2 in the colonization of the gut and internal organs of the chicken, we used a mixed infection approach [30]. We orally infected one-week old chickens with mixtures of two strains. Each strain carried different antibiotic resistance markers providing a simple means of identification. At days three, seven, and fourteen post-infection, groups of chickens were euthanized. The spleen and a sample of cecum from each bird were recovered, processed and plated for enumeration of colonies as described in the Methods section. The ratio of the two strains recovered from each organ was determined and compared to the input ratio to determine the competitive index (CI, ratio of the two strains from an organ divided by the ratio in the suspension used for the infection).

### In Vitro Testing of SPI1 and SPI2 Mutants

All strains containing SPI1 and SPI2 mutations were assayed for in vitro growth and invasion of the chicken macrophage cell line MQ-NSCU [31]. All mutants strains grew at approximately the same rate at the parent strain χ4138 (data not shown). Additionally, all mutants containing the Δ*spi1 *mutation were approximately thirty times less invasive than those with an intact SPI1 (data not shown)

### SPI1 contributes to the colonization of the cecum and of the spleen in chicken

**Figure 2:**
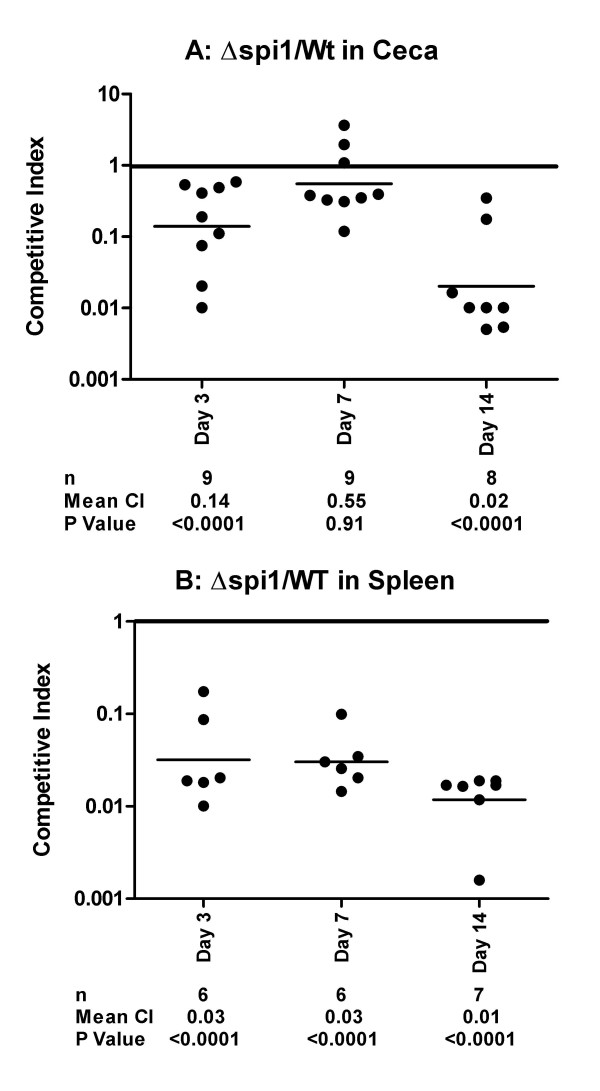
**Contribution of SPI1 to the colonization of chicken cecum (A) and spleen (B) by Typhimurium**. Competitive indexes are from mixed oral infections in chickens with the wild type and the Δ*spi1 *(deletion of SPI1) strains. Each point represents an organ from an individual bird at the indicated day following the infection. The table summarizes the number of animals sampled (n), the geometric mean of the competitive indexes (mean CI), and the P value from a two-tailed T-test.

In chickens infected with the wild type strain and its isogenic mutant lacking the entire SPI1 (Δ*spi1*), the Δ*spi1 *cells were significantly reduced in the ceca at days three (*P *< 0.0001) and fourteen (*P *< 0.0001) post-infection (Figure 2A). At day seven post-infection the difference between the two strains was not significant (Figure 2A).

Interestingly, the wild type out-competed the Δ*spi1 *strain in a more pronounced manner at day fourteen than at days three and seven post infection, suggesting an increased effect of the Δ*spi1 *mutation during long-term colonization of the cecum. For the spleen samples, the wild type out-competed the Δ*spi1 *strain in all the birds analyzed (Figure 2B) with the reduction of the Δ*spi1 *cells significant (*P *< 0.0001) at the three time points analyzed. Together these results show that SPI1 plays an important role in Typhimurium colonization of both the cecum and the spleen in chickens.

### SPI2 contributes to the colonization of the spleen but not of the cecum in one-week-old chickens

**Figure 3:**
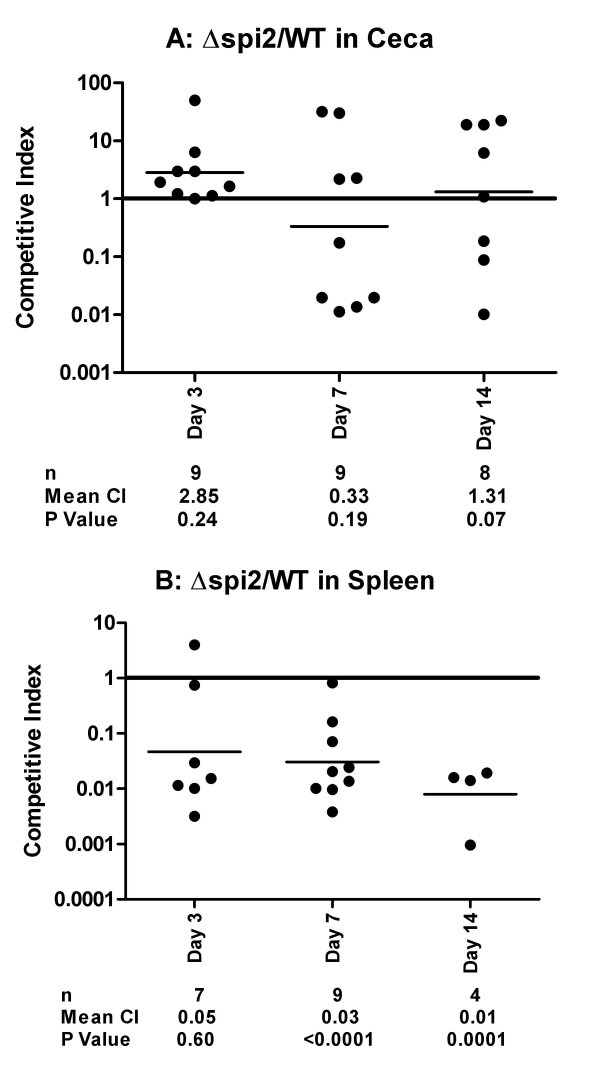
**Effect of Δ*spi2 *mutation (deletion of SPI2 structural genes) in the colonization of chicken cecum (A) and spleen (B) by Typhimurium**. Competitive indexes are from mixed oral infections in chickens with the wild type and the Δ*spi2 *strains. Each point represents an organ from an individual bird at the indicated day following the infection. The table summarizes the number of animals sampled (n), the geometric mean of the competitive indexes (mean CI), and the P value from a two-tailed T-test.

In the group of chickens infected with the wild-type and its isogenic mutant lacking the T3SS of SPI2 (Δ*spi2*), we did not observe significant differences, at any time point, in the cells recovered from cecal samples (Figure 3A). These results suggest that SPI2 does not contribute to the colonization of the chicken cecum by Typhimurium. To further test this hypothesis, we performed two co-infection experiments in which the effect of the Δ*spi2 *mutation was analyzed in the absence of SPI1. In the first experiment, we infected birds with a mixture of the wild type and the Δ*spi1 *Δ*spi2 *double mutant that lacks both SPI1 and SPI2 T3SS in order to test whether it differs from Δ*spi1 *with regards to the wild type.

**Figure 4:**
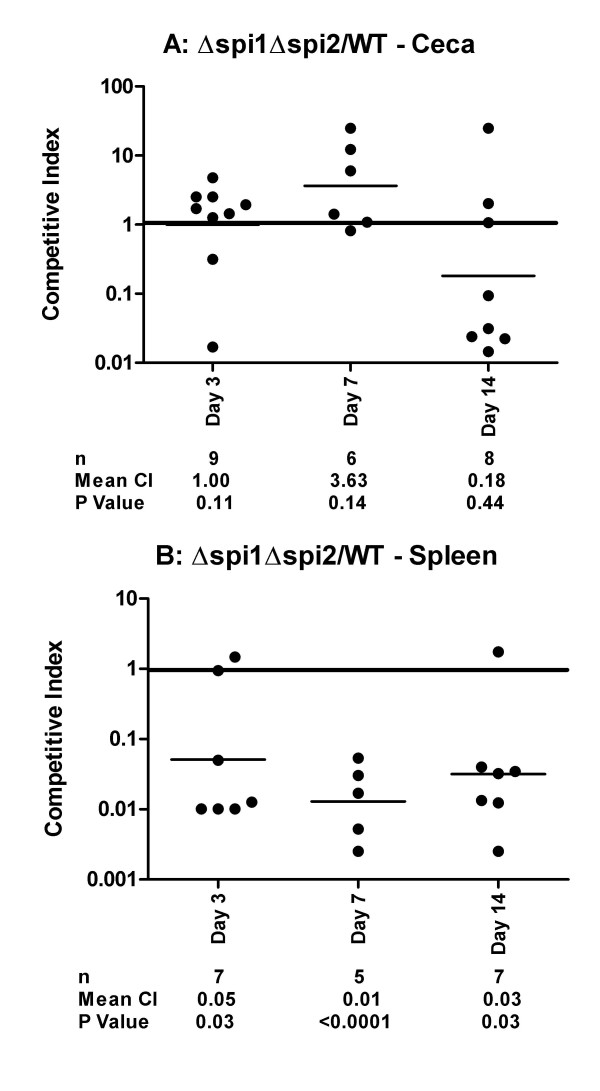
**Comparison of wild type and Δ*spi1 *Δ*spi2 *(deletion of SPI1 and the structural SPI2 genes) colonization of the chicken cecum (A) and spleen (B)**. Competitive indexes are from mixed oral infections in chickens with the wild type and the Δ*spi1 *Δ*spi2 *strains. Each point represents an organ from an individual bird at the indicated day following the infection. The table summarizes the number of animals sampled (n), the geometric mean of the competitive indexes (mean CI), and the P value from a two-tailed T-test.

**Figure 5:**
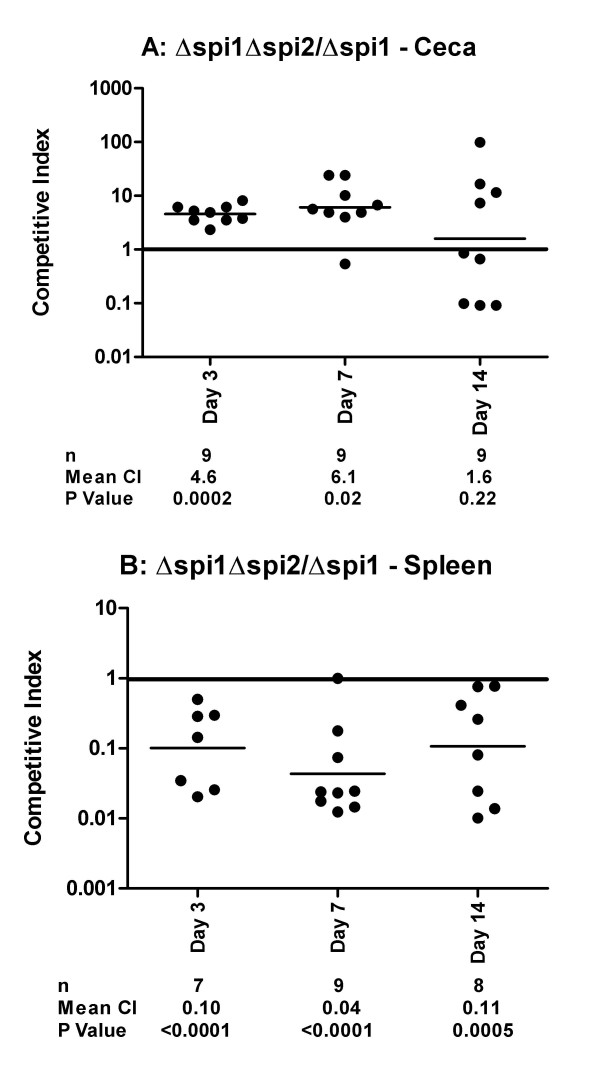
**Comparison of Δ*spi1 *Δ*spi2 *(deletion of SPI1 and the structural SPI2 genes) and Δ*spi1 *(deletion of SPI1) colonization of the chicken cecum (A) and spleen (B)**. Competitive indexes are from mixed oral infections in chickens with the Δ*spi1 *Δ*spi2 *and Δ*spi1 *strains. Each point represents an organ from an individual bird at the indicated day following the infection. The table summarizes the number of animals sampled (n), the geometric mean of the competitive indexes (mean CI), and the P value from a two-tailed T-test.

In the second experiment, we infected the chickens with a mixture of the Δ*spi1 *and the Δ*spi1 *Δ*spi2 *strains in order to verify whether the phenotype observed for the Δ*spi2 *strain in the mixed infection with the wild type is reproducible when SPI1 is absent in the two competing strains. There was no significant difference in the cells recovered from the ceca of the chickens infected with the wild type -Δ*spi1 *Δ*spi2 *mixture (Figure 4A). This is in direct contrast with the results from the wild type-Δ*spi1 *mixture (Figure 2A) and both confirms that the SPI2 T3SS is not required for colonization of chicken cecum by Typhimurium and suggests that the absence of SPI2 may have a positive influence on cecal colonization. Similarly, the Δ*spi1 *Δ*spi2 *strain significantly out-competed the Δ*spi1 *strain in cecal samples at days three and seven post infection (Figure 5A). This result is in direct contrast to that obtained from the wild type-Δ*spi2 *infection (Figure 3A) as when both strains are SPI1^+ ^there is no difference in cecal colonization. These results seem to suggest that the presence of the SPI2 T3SS negatively affects the colonization of the chicken cecum and that the presence of SPI1 tends to mask this phenotype. Altogether, these results both confirm that the SPI2 T3SS does not contribute to colonization of the chicken cecum by Typhimurium, and in SPI1^- ^strains actually inhibits cecal colonization.

In contrast to the observations from the cecal samples, SPI2^+ ^strains consistently and significantly out-competed isogenic SPI2^- ^strains in the spleen. This was observed when comparing the wild type and the Δ*spi2 *strain (Figure 3B), the wild type and the Δ*spi1 *Δ*spi2 *double mutant (Figure 4B), and the Δ*spi1 *and the Δ*spi1 *Δ*spi2 *strains (Figure 5B). Collectively, these results show that the SPI2 T3SS significantly contributes to the colonization of the spleen by Typhimurium in one-week-old chicks.

### SPI1 has a greater role than SPI2 in colonization of the spleen in one-week-old chicks

**Figure 6:**
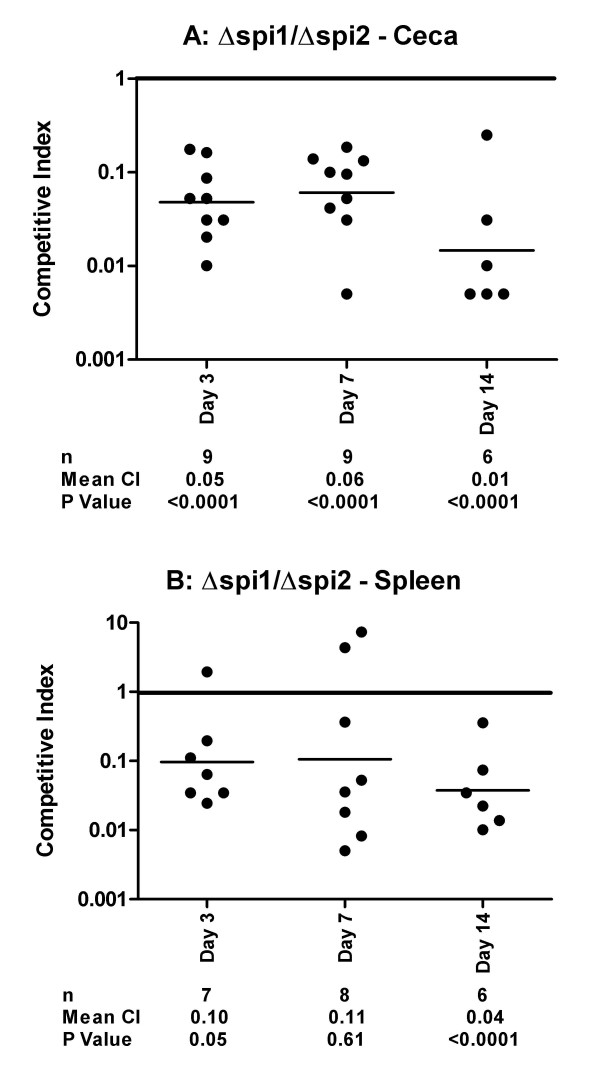
**Comparison of Δ*spi1 *(deletion of SPI1) and Δ*spi2 *(deletion of SPI2 structural genes) colonization of the chicken cecum (A) and spleen (B)**. Competitive indexes are from mixed oral infections in chickens with the Δ*spi1 *and Δ*spi2 *strains. Each point represents an organ from an individual bird at the indicated day following the infection. The table summarizes the number of animals sampled (n), the geometric mean of the competitive indexes (mean CI), and the P value from a two-tailed T-test.

Since SPI1 and SPI2 both contribute to splenic colonization and effect cecal colonization differently, we wanted to evaluate the relative importance of each of these virulence determinants. We infected chickens with a mixture of the Δ*spi1 *and Δ*spi2 *strains and found that the Δ*spi2 *strain significantly out-competed the Δ*spi1 *strain in the cecal samples (*P *< 0.0001) at days three, seven, and fourteen post-infection (Figure 6A). These results are consistent with the previous observation that SPI2^+ ^cells lacking SPI1 are significantly out-competed by SPI2^- ^bacteria (Figure 5A) and confirms that SPI1 (Figure 2A) but not SPI2 (Figures 3A, 4A, and 5A) contributes to cecal colonization.

**Figure 7:**
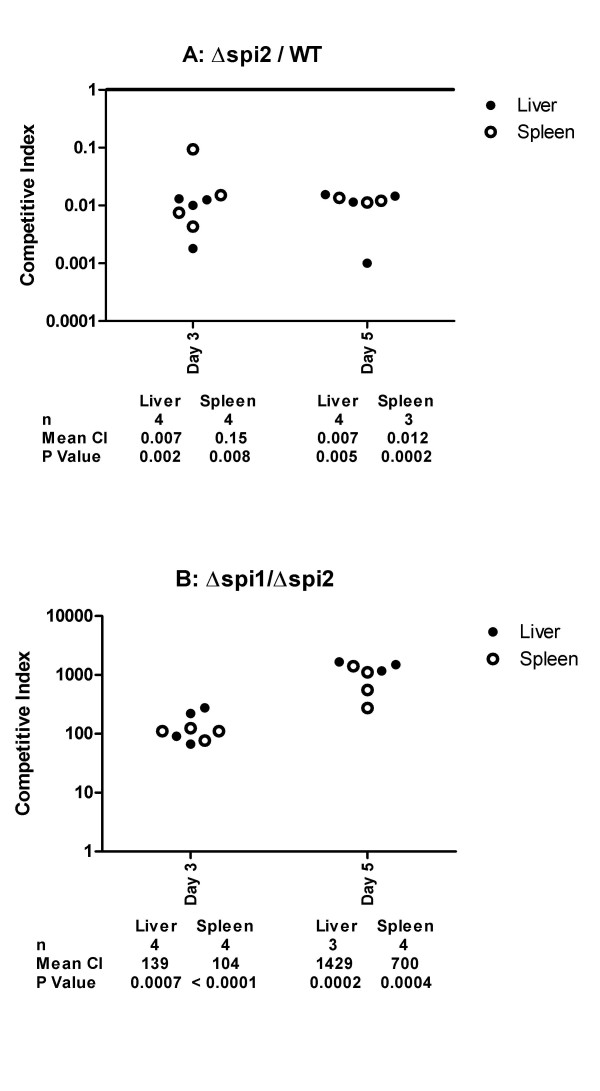
**SPI2 is essential to the colonization of mouse spleen by Typhimurium**. Competitive indexes are from mixed infections in mice with the wild type and the Δ*spi2 *(deletion of SPI2 structural genes), or the Δ*spi1 *(deletion of SPI1) and the Δ*spi2 *strains. Data from day 3 and day 5 post-infection correspond to intra-peritoneal and oral infections respectively. Each point represents an organ from an individual mouse.

Interestingly, the Δ*spi2 *strain also significantly out-competed by the Δ*spi1 *strain in the spleen at days three and fourteen post-infection (Figure 5B). This result suggests that SPI1 contributes more than SPI2 to splenic colonization. Since SPI2 has been shown in several animal models, including the mouse, to be a major factor for the survival of *Salmonella *in the systemic compartment of the host we decided to verify the accuracy of the results we obtained with the Δ*spi2 *strain in chicken spleen by performing mixed infection experiments in mice. As expected the Δ*spi2 *strain was out-competed by the wild type (Figure 7A) and the Δ*spi1 *strains (Figure 7B) in both the liver and spleen after either intra-peritoneal (Day 3) or oral (Day 5) infections. Collectively, these results show that in contrast to the mouse, SPI2 contributes less than SPI1 to splenic colonization of the chicken.

## Discussion

SPI1 and SPI2 are important virulence determinants of *S. enterica *serovars that have been extensively studied in several animal models. Few studies have investigated the role of SPI1 and SPI2 in the colonization of the chicken by Typhimurium. These studies have analyzed the colonization of different organs in chickens infected with a wild type strain or with mutants of SPI1 or SPI2 in which a single T3SS structural gene was inactivated. To gain better insight in the roles played by SPI1 and SPI2 in the chicken we used an approach that combined mixed infections, large deletions in SPI1 and SPI2, and the tracking of infections for fourteen days. We found that SPI1 contributes to colonization of both the cecum and the spleen in chickens. In contrast, SPI2 plays a role in the colonization of the spleen, but not of the cecum. Furthermore, we show for the first time to our knowledge, that SPI1 plays a more important role than SPI2 in colonization of the chicken spleen by Typhimurium. We cannot exclude the possibility that the phenotypes conferred by the mutations we constructed resulted from pleiotropic effects given that we deleted the entire SPI1 and the majority of SPI2 T3SS genes, and that SPI1 and SPI2 regulators have been shown to act on the expression of genes located outside these pathogenicity islands whose functions are unrelated to T3SS [24,32]. This has been reported previously in mice where the deletion of the entire SPI1 had a different effect than a single gene deletion [33]. However, it seems unlikely as other studies have yielded results that are consistent with some of our findings. For instance, two studies that screened transposon mutant libraries of Typhimurium for reduced colonization of the chicken gastrointestinal tract either found mutations in SPI1 but not in SPI2 [28] or that SPI1 mutations had greater influence [29]. Despite the fact that cecal swabbing was used to recover strains in these two studies, which may fail to catch low level colonization, both studies still identified SPI1 as important in intestinal colonization. Cecal colonization was also reported to decrease substantially after the deletion of SPI1 T3SS components [26]. Additionally, a study with *S. enterica *serovar Enteritidis, which displays an infection pattern similar to Typhimurium, showed that deletion of the *ssrA *gene, encoding the sensor component of the SsrAB two-component system that is the major regulator of the SPI2 gene expression, did not affect the colonization of the chicken digestive tract [34]. All together these results suggest that Typhimurium relies less on SPI2 than on SPI1 for colonization of the intestinal track in one-week-old chicks.

In contrast, Jones et *al*. [27] analyzed the contribution of SPI1 and SPI2 to the colonization of chickens by Typhimurium through the deletion of a single T3SS structural gene in each. They concluded that the SPI2 T3SS was required for systemic infection and played a significant role in the colonization of the gastrointestinal tract, while the SPI1 T3SS was involved in both compartments without being essential [27].

There are several important differences between that study and ours. First, Jones *et al*. used derivatives of the Typhimurium F98 strain [9] while we used derivatives of the UK-1 strain [36]. While both have been well characterized for virulence and persistence in chickens, their mean lethal dose (LD_50_) in day of hatch chicks differ by two orders of magnitude with F98 at 5 × 10^5 ^cfu [35] and UK-1 at approximately 2 × 10^3 ^[36]. Second, they studied mutants in which a single structural T3SS gene was inactivated while in our mutants the entire SPI1 and all the SPI2 T3SS structural genes were deleted. Third, they determined the level of colonization of the chicken by calculating the bacterial density (number of colony forming unit per gram) in the organs after administration of single strains while we infected the chickens with mixtures of the two strains being compared and determined the competitive index. These differences may account for the differences in the results.

Through the use of different combinations of mutants used for infection we have observed that strains harboring the Δ*spi2 *mutation have a modest advantage in the colonization of the chicken cecum, and therefore SPI2 may act to repress some factor needed for cecal colonization. However, this observation was only statistically significant when SPI1 was absent both in the strain that harbored the Δ*spi2 *mutation and the competing strain (Figure 5A). We have come to this conclusion based on the above observation in addition to the fact that while the Δ*spi1 *is out-competed by the wild type (Figure 2A), the double mutant Δ*spi1 *Δ*spi2 *is not (Figure 4A). We do not know the basis of this disadvantage conferred by the presence of SPI2 in the colonization of chicken cecum by Typhimurium. One explanation is that genes deleted from SPI2 may normally act to repress some factor needed for the colonization of the cecum but in their absence this factor is not repressed, thus increasing invasion. An alternative explanation may be that the phenotype conferred by the Δ*spi2 *mutation in not decreasing intestinal colonization results from the absence of SPI1 regulators, such as HilD, that are known to regulate SPI2 genes, including the SsrAB central regulator. Additional investigations are needed to test these hypotheses.

In contrast to what we have observed in chickens, SPI2 is the major contributor for spleen colonization in BALB/c mice. The infection by Typhimurium in these two animal models leads to different outcomes. In mice, Typhimurium causes an acute systemic infection, frequently resulting in death, while in one-week or older chickens, the infection leads to heavy colonization of the intestinal track and asymptomatic carriage. It is interesting to note that in animal models where *Salmonella *infection results in acute systemic disease, SPI2 is a major player in the systemic infection. These include the infection of mice by Typhimurium [12], and the systemic disease in chickens infected by serovars Pullorum [37] and Gallinarum [38]. In contrast, in animals where infection results in healthy carriage, such as in chickens, SPI2 plays a minor role in the persistence of the bacteria in the systemic compartment. This is demonstrated in the present study, and has been reported for Typhimurium in pigs [39], and for serovar Enteritidis in chicken [40]. This difference in contribution of SPI2 in these two situations indicates that SPI2 is an important factor of *Salmonella *host specificity.

## Conclusion

We have taken a mixed infection approach to study the role of SPI1 and SPI2 in the colonization of the chicken by Typhimurium. We confirmed the contribution of SPI1 to the colonization of both the cecum and the spleen, and showed that SPI2 is involved in the colonization of the spleen but not of the cecum and, may have a negative effect on cecal colonization. Additionally, we show that SPI1 plays a greater role than SPI2 in the colonization of the spleen in chickens. In contrast, SPI2 is more important than SPI1 for systemic colonization in mice. The approach we used in this study constitutes a sensitive assay that provided new insights into the role of SPI1 and SPI2 during infection.

## Methods

### Bacterial growth, enzymes, reagents, and transduction

The bacterial strains were grown in LB broth [41] or on LB plates at 37°C. The following antibiotics were obtained from Sigma and used at the following concentrations when required: kanamycin (Km), 50 μg/ml, ampicillin, 100 μg/ml, chloramphenicol (Cm), 20 μg/ml, nalidixic acid (Nal), 30 μg/ml.

General molecular biology techniques were performed essentially as described [42]. Restriction and modification enzymes were purchased from Invitrogen (Carlsbad, CA) or New England Biolabs (Beverly, MA), and used as recommended by the manufacturers. PCR primers were purchased from IDT Inc. (Coralville, IA). P22 transduction was performed as described [43].

### Strains

The following Typhimurium strains, that are derivatives of the UK-1 wild-type strain, were constructed and used in this study. (I) The SPI1^+^SPI2^+ ^strain χ4138, *gyrA1816*, Nal^R^. (II) The SPI1^-^SPI2^+ ^(Δ*spi1*) strain *χ*9648 *gyrA1816 *Δ(*avrA-invH*)-*2*::*cat*, Nal^R^, Cm^R^. (III) The SPI1^+^SPI2^- ^(Δ*spi2*) strain, χ9649 *gyrA1816 *Δ(*ssaG-ssaU*)-*1*::*kan*, Nal^R^, Km^R^. (IV) The SPI1^-^SPI2^- ^(Δ*spi1 *Δ*spi2*) strain χ9650 *gyrA1816 *Δ(*avrA-invH*)-*2*::*cat *Δ(*ssaG-ssaU*)-*1*::*kan*, Nal^R^, Cm^R^, Km^R^.

### Strain construction

The χ4138 strain was made by P22-mediated transduction of the *gyrA *mutation from χ3147 [44] into the wild-type UK-1 strain χ3761, selecting for nalidixic acid resistance.

The mutations in SPI1 and SPI2 were constructed in strain JS246 [45] using the λ-red recombination system [46]. The deletion of the T3SS genes of SPI1 was performed using a PCR fragment obtained with the primers YD142 (5'gctggaaggatttcctctggcaggcaaccttataatttca**gtgtaggctggagctgcttc**3') and YD143 (5'taattatatcatgatgagttcagccaacggtgatatggcc**catatgaatatcctccttag**3'). YD142 harbors 40 nucleotides that bind downstream of the stop codon of the *avrA *gene, and 20 nucleotides (in bold) that correspond to PS1 [46]. YD143 harbors 40 nucleotides that bind downstream of the *invH *gene, and 20 nucleotides (in bold) that correspond to PS2 [46]. The T3SS2 structural genes of SPI2 were deleted using a PCR fragment obtained with the primers SPI2a

(5'gctggctcaggtaacgccagaacaacgtgcgccggagtaa**gtgtaggctggagctgcttc**3') and SPI2b (5'tcaagcactgctctatacgctattaccctcttaaccttcg**catatgaatatcctccttag**3'). SPI2a harbors 40 nucleotides that bind upstream of the *ssaG *gene, and 20 nucleotides (in bold) that correspond to PS1. SPI2b harbors 40 nucleotides that bind at the end of the *ssaU *gene, and 20 nucleotides (in bold) that correspond to PS2. The deletions were verified by PCR from the genomic DNA using the appropriate primers. The Δ*spi1 *and Δ*spi2 *mutations were introduced into χ4138 by P22-mediated transduction to construct χ9648 and χ9649, respectively. χ9650 was constructed by transducing the Δ*spi1 *mutation into χ9649. All mutant strains were assayed for in vitro growth rate and were comparable to the wild type (data not shown), as well as tested for invasion in the macrophage cell line MQ-NSCU [31]. All strains containing the Δ*spi1 *mutation were approximately thirty times less invasive than those with an intact SPI1 (data not shown).

### Animal Infection

All the animal experiments were conducted in accordance with protocols approved by the Arizona State University Institutional Animal Care and Use Committee. Specific-pathogen-free fertile white leghorn eggs were obtained from SPAFAS Inc. (Roanoke, IL.) and hatched at the animal facilities of the Biodesign Institute, Arizona State University. At hatching, chicks were placed into isolators equipped with HEPA filters. The bacterial strains were grown to an OD_600 _of ~0.8. Equal volumes of cultures of strains that were co-administered were mixed and centrifuged at 4,000 × g at room temperature. The cells were then suspended in phosphate-buffered saline containing 0.01% gelatin to a final concentration of approximately 2 × 10^10 ^CFU/ml. Dilutions of this suspension were plated onto LB plates containing the appropriate antibiotics for the determination of the density and of the ratio of the strains from each mixture. For the infections, one-week-old chickens were deprived of food and water for 6 h prior to bacterial administration. 50 μl of bacterial suspension corresponding approximately to 10^9 ^CFU were orally administered to chickens. Food and water were returned to the birds 30 minutes after infection.

Female six week old BALB/c mice (Charles River Laboratories, Wilmington, MA) were fasted for food and water for six hours before oral infection with 20 μl of bacterial suspension (~10^9 ^CFU) prepared as described above. Food and drink were returned 30 minutes after infection. For intra-peritoneal infection mice were injected with 100 of bacterial suspension containing 10^3^–10^5 ^CFU.

### Organ processing

All animals were euthanized by asphyxiation with CO_2_. The spleen and an approximately 3 cm piece of the cecal pouch (wall and content) were aseptically taken from each bird and homogenized (PowerGen 125 S1, Fischer Scientific, Pittsburgh, PA) in PBS. The spleen, or the spleen and a piece of the liver were recovered aseptically from each mouse and homogenized. Dilutions of these samples were plated onto McConkey-1% lactose (MC) plates containing the appropriate antibiotics. Samples from animals infected with χ4138 and χ9648, χ4138 and χ9649, χ4138 and χ9650, and χ9648 and χ9648 were plated onto MC-Nal and MC-Nal-Cm, MC-Nal and MC-Nal-Km, MC-Nal and MC-Nal-Cm, and MC-Nal, MC-Nal-Cm and MC-Nal-Km plates, respectively. The ratios of the strains recovered from the organs were determined by enumerating the colonies on the different plates and by patching colonies from MC-Nal plates onto plates containing the appropriate antibiotics.

### Competitive index and statistical analysis

The competitive index is given by dividing the ratio of two strains from an organ divided by the same ratio in the suspension used for the infection. The geometric means of the CIs were determined and a Student's *t*-test was used to determine whether the logarithmically transformed ratios differed significantly from 0. A *P *value below 0.05 was considered statistically significant.

## Authors' contributions

RCIII provided the idea for this study. YD designed the experiments and constructed the mutants. YD, KA, and MM performed the animal experiments. YD wrote the manuscript.

RCIII and KA revised the manuscript. All authors read and approved the final manuscript.
